# 

**Published:** 1886-02

**Authors:** 


Entered at the Post Office, at Austin, Texas, as Second-Class Matter.

VOL. 1.] FEBRUARY, 1886. [NO. 8.

LIBRARY
INDEX
S.G.C.

### DANIEL'S MEDICAL JOURNAL

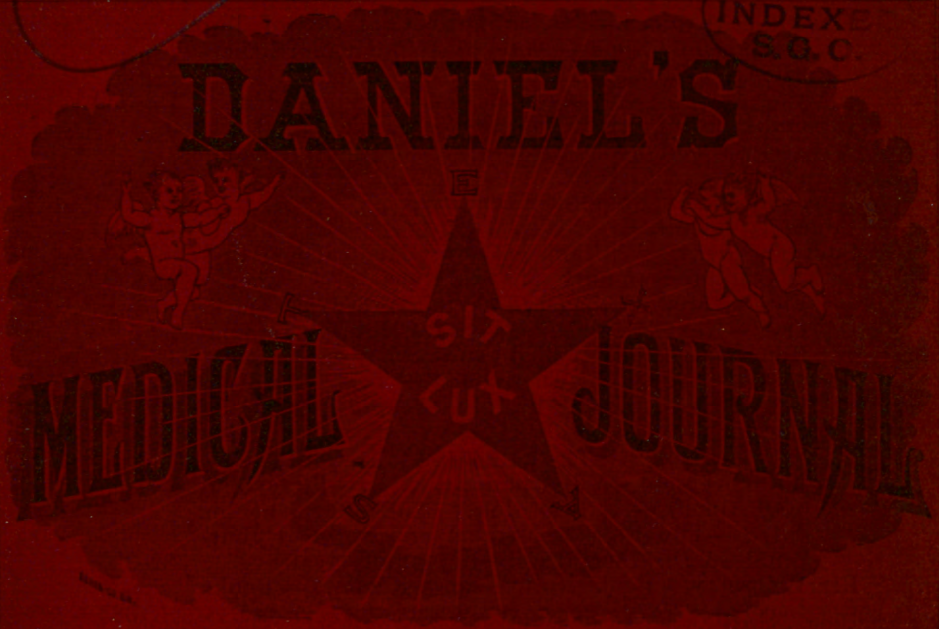

*Subscription: $2.00 a Year in Advance; - - - Single Copies, 25 Cents.*

F. E. DANIEL, M. D., Editor and Publisher, AUSTIN, TEXAS.
NORTHERN OFFICE, 1217 FILBERT STREET, PHILADELPHIA.

*EUGENE VON BOECKMANN, PRINTER, AUSTIN, TEXAS.*

